# Altered expression pattern of circular RNAs in primary and metastatic sites of epithelial ovarian carcinoma

**DOI:** 10.18632/oncotarget.8917

**Published:** 2016-04-22

**Authors:** Ikhlak Ahmed, Thasni Karedath, Simeon S. Andrews, Iman K. Al, Yasmin Ali Mohamoud, Denis Querleu, Arash Rafii, Joel A. Malek

**Affiliations:** ^1^ Department of Genetic medicine, Weill Cornell Medicine-Qatar, Education City, Ar-Rayyan, Qatar; ^2^ Genomics Core, Weill Cornell Medicine-Qatar, Education City, Ar-Rayyan, Qatar; ^3^ Department of Gynecologic Oncology, Université Montepllier 1, Montpellier, France; ^4^ Stem Cell and Microenvironment Laboratory, Weill Cornell Medicine-Qatar, Education City, Ar-Rayyan, Qatar

**Keywords:** circular RNA, ovarian cancer, peritoneal metastasis, lymph node metastasis, miRNA

## Abstract

Recently, a class of endogenous species of RNA called circular RNA (circRNA) has been shown to regulate gene expression in mammals and their role in cellular function is just beginning to be understood. To investigate the role of circRNAs in ovarian cancer, we performed paired-end RNA sequencing of primary sites, peritoneal and lymph node metastases from three patients with stage IIIC ovarian cancer. We developed an in-house computational pipeline to identify and characterize the circRNA expression from paired-end RNA-Seq libraries. This pipeline revealed thousands of circular isoforms in Epithelial Ovarian Carcinoma (EOC). These circRNAs are enriched for potentially effective miRNA seed matches. A significantly larger number of circRNAs are differentially expressed between tumor sites than mRNAs. Circular and linear expression exhibits an inverse trend for many cancer related pathways and signaling pathways like NFkB, PI3k/AKT and TGF-β typically activated for mRNA in metastases are inhibited for circRNA expression. Further, circRNAs show a more robust expression pattern across patients than mRNA forms indicating their suitability as biomarkers in highly heterogeneous cancer transcriptomes. The consistency of circular RNA expression may offer new candidates for cancer treatment and prognosis.

## INTRODUCTION

Epithelial ovarian carcinoma (EOC) is the fifth most common cancer among women and one of the most lethal gynecological tumors, being responsible for more deaths than any other cancer of the female reproductive system [1]. Most patients are diagnosed at advanced stage with extensive peritoneal metastasis, resulting in a five year survival rate of only 30% [2]. Lymph nodes are increasingly involved as the tumor spreads through the intraperitoneal route in advanced disease stages [3]. Disease recurrence and poor prognosis remains a problem despite initial chemosensitivity and enhanced surgical procedures [4, 5].

Current tumor models fail to reflect their outcomes when applied to clinical samples, as the intra-tumor heterogeneity of multiple genotypes and phenotypes is a major obstacle to robust, cross-patient analysis. The tumors represent a complex ecosystem composed of cells with distinct phenotypes, genotypes and epigenetic states which co-exist and evolve simultaneously [6]. Conventional therapies against cancer often lead to resistance, resulting in tumor relapse. Personalized therapies based on characterization of the most abundant clones in the tumor mass may not accurately predict the total properties of that tumor or the best treatment regimen. It is therefore essential to accurately measure the genetic and molecular signatures of a tumor in order to decide upon a proper personalized chemotherapy strategy.

We have previously described copy number variation (CNV) and gene expression analysis of matched ovarian primary tumors and peritoneal metastases, and reported targeting of several specific pathways that play a role in ovarian cancer metastasis both at genomic and transcriptomic levels [7, 8]. Many other studies have also focused on delineating the gene expression signatures for disease prognosis and therapeutic responses [9–11]. However, the robustness and reproducibility of these molecular signatures is yet to be clearly established owing to a highly heterogeneous nature of the tumor sample. The relationship between different sources of this heterogeneity remains elusive and includes both fluctuating transcriptional signal and stochastic genetic variation of tumor mass. Therefore, improved markers and therapeutic targets that are less prone to tumor heterogeneity are needed for better prognostic and therapeutic results.

Recently, a novel class of RNA termed circular RNAs (circRNA) is increasingly being recognized as an abundant class of regulatory transcripts primarily derived from protein coding exons and widely expressed across eukaryotic organisms including *Homo sapiens* and *Mus musculus* [12–17]. These circularized transcripts have been shown to be produced co-transcriptionally by the spliceosome at the expense of canonical mRNA isoforms [18] in a process in which exons are atypically joined in a non-linear order through a head-to-tail “backsplice” junction formation. The exons forming these “backsplices” are often flanked by longer introns enriched in ALU repeats and the circularization is dependent on flanking complementary sequences [14, 19, 20]. The circularization efficiency of backsplice forming exons seems to be regulated by their flanking introns through competition for RNA pairing between complementary sequences [19]. These intronic sequences promoting circularization therefore appear to modulate linear splicing such that circRNA production and linear splicing mutually regulate each other by competing for splice sites. Therefore the mere production of circRNAs has been proposed to have a regulatory impact on host mRNA [18]. Further, circRNAs have also been shown to function as competing endogenous agents that act as decoys for the binding of miRNA. Thus they act as sponges of miRNA, regulating the expression of miRNA targets. Indeed, circRNAs from *CDR1* and *Sry* genes have been shown to bind miR-7 and miR-138, respectively, at multiple binding sites without getting degraded, making them excellent candidates for competing endogenous RNA activity [15, 21, 22]. Thus circRNAs can offer effective ways for the use of these sponge elements as potential therapeutic agents to target oncogenic miRNAs [23, 24] or provide biomarkers with diagnostic and prognostic abilities.

To investigate the role of circRNAs in ovarian cancer, we performed paired-end RNA sequencing of nine ovarian cancer samples from three patients at primary ovarian tumor and its matched peritoneum and lymph node metastatic stages. We developed an in-house computational pipeline to identify and characterize the circRNA expression from paired-end RNA-seq data. Our results show that circRNAs are widely expressed in epithelial ovarian cancer with thousands of circular isoforms present in primary ovarian tumor and its peritoneum and lymph node metastases. These candidate circRNAs are enriched for miRNA seed matches and potentially capable for competing endogenous RNA activity. Moreover, as against a highly heterogeneous linear transcriptome in ovarian cancer, we report a robust expression pattern of circRNAs across patients and tumor stages.

## RESULTS

### The repertoire of circRNA candidates in ovarian cancer

**Figure 1:**
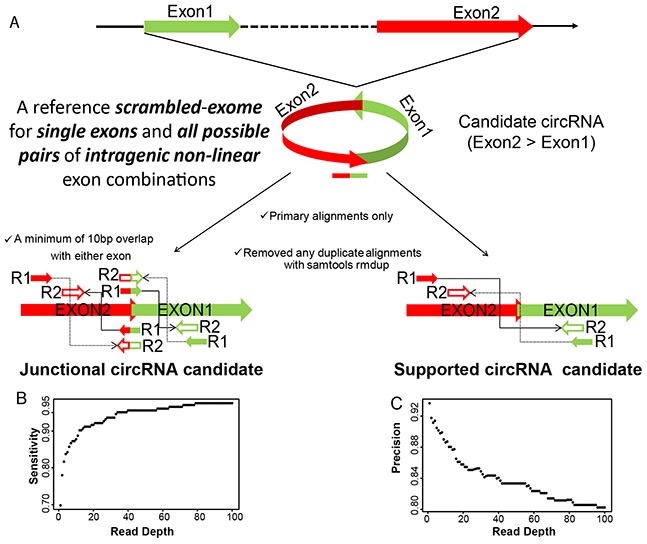
**A. circRNA detection pipeline.** circRNA candidates are inferred from alignment of paired-end RNA-seq data to a reference scrambled exome. Read-pair alignments that contain apparent backsplice junction are reported as either junctional or supportive evidence for the backspliced exon junctions. **B. and C. Sensitivity and Precision of circRNA detection pipeline.** Simulated reads at different read depths representing the expression level of randomly selected backsplice junctions mixed with a background of their canonical transcripts were run through the detection pipeline. Measurements of the correctly called circRNA junctions (true positives;TP), incorrectly called junctions (false positives, FP) and unidentified junctions (false negatives, FN) were used to define sensitivity and precision as TP/(TP+FN) and TP/(TP+FP), respectively.

Total RNA was collected from the biopsies of the primary tumor, as well as peritoneum and lymph node metastases of three ovarian cancer patients presenting with stage IIIc papillary serous adenocarcinomas [8]. Tumor samples contained at least 80% of tumor cells and displayed less than 20% necrosis. RNA was extracted with commercially available kits and prepared for Illumina sequencing by using Encore Complete library preparation kits (Nugen), which produce very low levels of ribosomal RNA reads and also amplifies non-polyA containing transcripts. Illumina deep sequencing yielded an average of 46.96 million read pairs per library of 46-50 bp read length. Of these around 66-72% read pairs mapped concordantly to the linear genome, while 32-37% of the pairs concordantly mapped to a database of all possible non-linear backspliced exon junctions and were used to infer circRNA candidates. The biogenesis of circRNAs represents a non-canonical mode of RNA splicing in which a downstream exon (Exon2) is “backspliced” in a head-to-tail fashion to an upstream exon (Exon1) resulting in a circular RNA molecule placing Exon2 upstream of Exon1. The circular RNAs can also form when the splicing machinery joins the two ends of a single exon via the process of “backsplicing”. We created a reference scrambled exome for all possible pairs of intragenic non-linear combinations of exons, as well as single exon “backsplices”, representing the sample space for circular junctions and aligned the RNA-seq data to this reference scrambled exome with Bowtie2 [25]. These alignments were further filtered to remove any potential PCR duplicates and only primary alignments were kept for final assessment of circRNA expression (Figure 1A). Finally from these alignments, a scrambled junction is inferred as a junctional circRNA candidate when one mate of a paired-end read aligns at the junction with a minimum of 10bp overlap with either exon and the other mate aligns at either Exon2 or Exon1. Alternatively, in absence of a direct junctional read, supportive evidence is also considered when mates of a paired-end read align to the exons in divergent orientation with respect to the genomic sequence, suggesting a scrambled junction instead of a linear junction (Figure 1A).

We assessed the extent of alignment artifacts or DNA sequence redundancies towards the backsplice junction identification process and therefore any inadvertent discovery of circRNA candidates. To this end, we first randomized the DNA sequence of each of the exons for all exon pairs used in our circRNA detection pipeline, and aligned the randomized exome to the paired end RNA-seq data to look for any backsplice junctions, and as expected no junction was found. To make the DNA sequence less random, in a second step only the downstream exon (Exon2) was flipped without randomizing the DNA sequence and again aligned to the paired end RNA-seq data to look for any head-to-head spliced (Exon1-flipExon2) junctions, we found only 33 such junctional events having zero overlap with our circRNA candidates. This convincingly indicates that mis-alignments and DNA sequence redundancies have been genuinely ruled out and do not contribute towards candidate circRNA identification process. To estimate the sensitivity and precision of our circRNA detection pipeline, we applied it to a simulated dataset of 50bp paired-end reads for ~200 randomly selected “backsplices” mixed with a background of their linear canonical transcripts generated using mason software [26]. Figure 1B and C evaluates the sensitivity and precision values of our circRNA detection pipeline as a function of read depth and indicates a remarkably better performance of our pipeline as against other published softwares [27]. The successful mapping to the backsplice exon junctions inferred as candidate circRNAs in this study resulted in a total of 67580 unique circRNA candidates across the nine tumor samples, with either at least three independent junctional read pairs or ten supporting read pairs for the “backsplice” junction (Supplementary File S2, Table 1). This represents only 8.5% of the initially identified “backsplices”, the majority of which did not satisfy our criteria of three junctional or ten supported read pairs (Supplementary Figure S1). We therefore discarded more than 90% of the initially identified candidates having less than three junctional or ten additional read pairs supporting the non-linear backsplice exon junction, as they may represent artifacts of template switching from reverse transcription [28–30], chimeric amplification [31, 32] or trans-splicing [33, 34], which are rare and random events, not expected to produce abundant products. Of note, this also excludes the well-characterized single-exon circRNA from CDR1-AS [15], which had only three backsplice supporting read pairs in our data, but no junctional reads.

**Table 1:** Number of identified Junctional and supported circRNA candidates for clinical ovarian primary tumor and its peritoneal and lymph node metastases for three ovarian cancer patients. Junctional candidates have backsplice junction spanning reads in at least one of the nine tumor samples

Patient	Ovary		Peritoneum		Lymph node	
	Junctional	Supported	Junctional	Supported	Junctional	Supported
Ptnt1	19990	12988	25179	16932	23840	15593
Ptnt2	20352	13176	25014	16013	23566	15726
Ptnt3	29736	20234	24681	16497	25913	17650

### Validation of circRNA candidates

**Figure 2:**
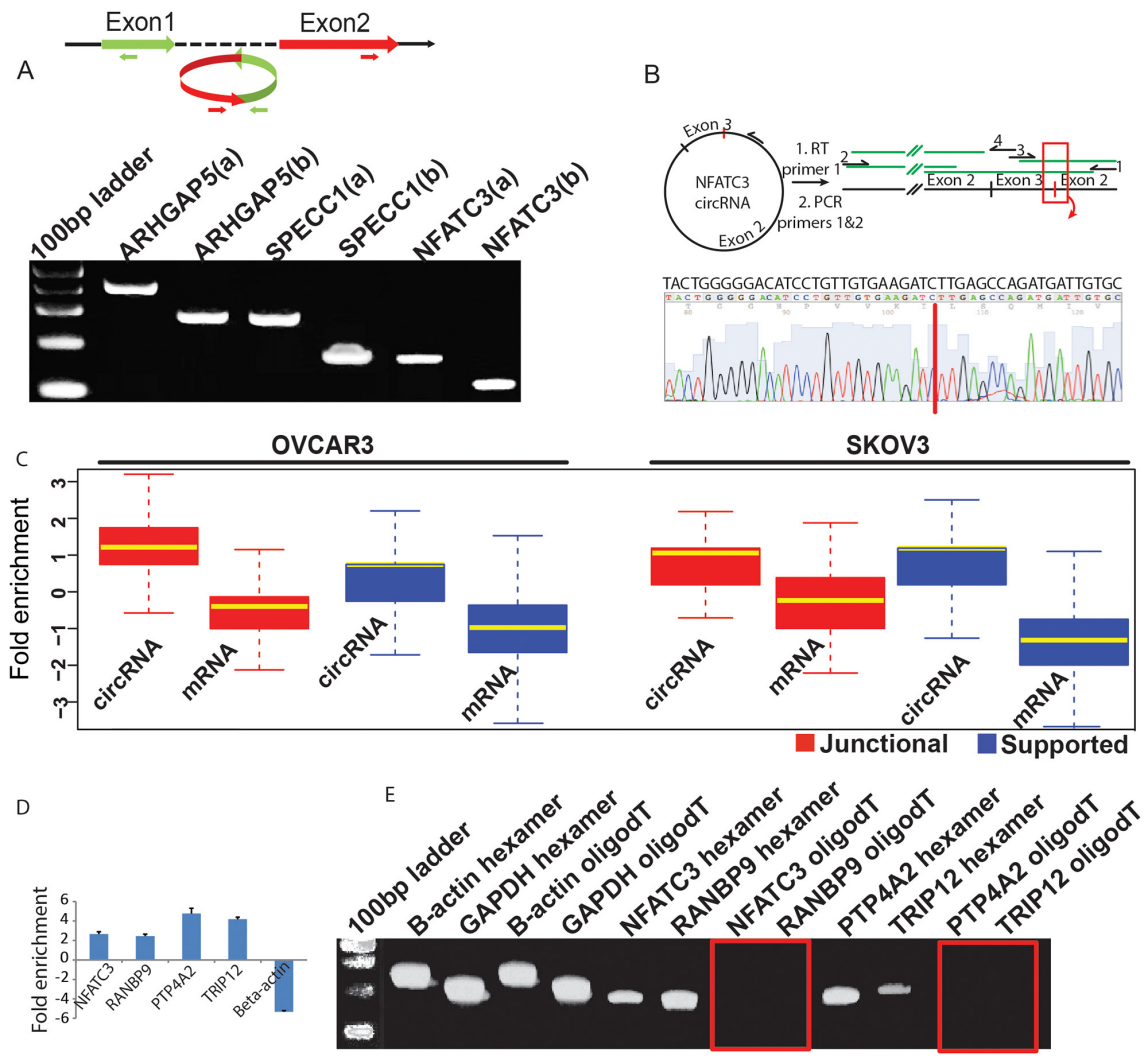
**A. Divergent primers become properly inward facing and identify backspliced junction for circRNAs.** Two sets of outward facing primers (a & b) with respect to genomic sequence were designed to amplify the backsplice junction sequence for circRNA candidates from ARHGAP5, SPECC1 and NFATC3 genes and each produced an expected size band in the qPCR assay (Supplementary File S1). **B. Sanger sequencing confirms head-to-tail splicing for NFATC3.** RNA samples were reverse transcribed with thermo-stable reverse transcriptase and a primer complementary to the coding RNA strand. PCR on the cDNA produced with primers 1 and 2 resulted in large amounts of product that could be Sanger sequenced. Primers 1 to 4 were used in sequencing, with coverage from each primer indicated in green. Expansion of the sequence provided by primer 3 shows complete coverage of the non-canonical junction. **C. circRNA candidates are enriched after the RNase R digestion.** Total RNA from OVCAR3 and SKOV3 cell lines was treated with RNAse R to test the sensitivity of the identified circRNA candidates to exoribonuclease digestion. Boxplots represent FPKM (Fragments per kilobase of exons per million mapped fragments) fold change for RNAse R treated vs. untreated samples and shows a significant enrichment of both junctional and supported circRNAs (n=7903) compared to mRNA (n=2785) in the RNAse R digested samples. Only genes that produced at least one detectable circular isoform were considered for this analysis. **D. qPCR confirms RNAse R enrichment for circular junctions.** Total RNA from OVCAR3 cell line was first treated with DNase1 to avoid any DNA contamination and then part of it was subjected to the RNase R digestion followed by RT-PCR for each sample. The four putative circular RNAs show enrichment for the backsplice junction compared to the linear RNA from beta-actin used as a negative control. **E. Candidate circRNAs are devoid of Poly-A tails.** The four candidate circRNAs were also tested for the presence /absence of a ploy-A tail by using either oligo (dT) or random hexamers to amplify the total RNA followed by a qPCR assay with primers specific for backsplice junctions of candidate circRNAs and linear RNA of b-actin and GAPDH genes. While both oligo (dT) and random hexamers could amplify the RNA from beta-actin and GAPDH, only random hexamers showed detectable PCR products for the four tested candidate circRNAs. The red rectangles show absence of any PCR bands for the backsplice junctions.

In order to validate that observed circRNA candidates are not the misidentified linear products of trans-splicing events [32], we designed multiple pairs of outward facing primers for representative circRNA candidates (Supplementary File S1) to amplify the backsplice exon junction from cDNA of the ovarian cancer cell line NIH:OVCAR-3. Each primer pair indeed produced a single distinct band of expected product size in an RT-PCR assay, indicating the presence of the circular junction in a cell-line RNA sample (Figure 2A, 2B). Further evidence of a circularized structure for the circRNA candidates came from the digestion of total RNA from OVCAR3 and SKOV3 cell lines with an exoribonuclease enzyme RNase R, followed by high throughput RNA-sequencing for both RNAse R treated and untreated samples. This exonuclease enzyme digests all linear RNA forms with a 3′ single stranded region of greater than 7 nucleotides [35]. As circRNAs are devoid of any 3′ single strand overhangs, they are expected to show resistance to the digestion by RNase R. Indeed, there was an enrichment of circRNA candidates after the RNase R treatment compared to mRNA confirming the resistance of the identified circRNA candidates to exoribonuclease digestion (Figure 2C, 2D). Of the 67580 circRNA candidates identified in patient samples, 34730 (51.4%) were also identified in RNase R treated libraries of the two cell lines indicating a significant overlap (p-value < 0.00001) and corroborating the high sensitivity and precision values of our circRNA detection pipeline. Furthermore, when cDNA was created by priming with oligo (dT) primers, it is expected to amplify only poly-adenylated RNAs. Indeed, this cDNA failed to produce any amplification products for the circRNA candidates in the RT-PCR assay. On the other hand, priming with random hexamers – which can also amplify non poly-adenylated RNAs – resulted in distinct PCR bands of backsplice junctions. This strongly indicates the absence of a ploy-A tail for the candidate circRNAs (Figure 2E).

### Diversity and relative abundance of candidate circRNA isoforms in ovarian cancer

**Figure 3:**
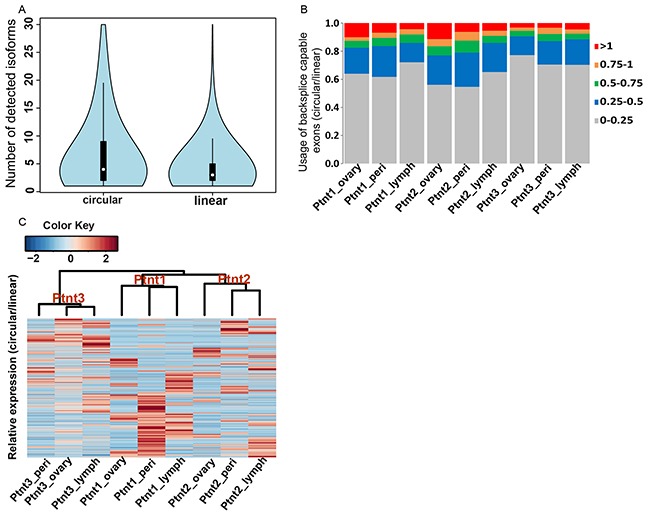
**A. Isoform diversity of candidate circRNA versus linear transcripts.** Violin plots - a combination of boxplot and kernel density function compares the abundance levels of candidate circRNA isoforms and cufflinks assembled linear transcripts for 7121 genes for which at least one detectable backsplice junction was observed. The diversity of candidate circRNA isoforms is significantly higher (wilcoxon test; p-value 3.324e-07) than that of linear transcripts in ovarian cancer. **B. Usage of backsplice capable exons as linear and circular forms.** For the backsplice junction forming exons, ratio of read pairs supporting the scrambled form to the read pairs supporting the linear form defines the relative abundance of candidate circRNA expression. Most backsplice capable exons are relatively more abundant in the linear form, however a significant proportion (5-10%) of these are preferentially expressed as circRNA form. Colors indicate ratio of circular to linear expression. **C. Heatplot of relative abundance levels.** Each line in the plot is a circRNA candidate as defined by two backsplice capable exons with colors indicating relative abundance levels of circular over linear expression for the two exons. Hierarchically clustered heatmap of the relative abundance levels shows the clear trends of stronger intra-patient homogeneity of circRNA expression as indicated by a distinct dendrogram branch for each patient. Sub-branching for patients 1 & 2 further reveals a primary tumor and metastases specific expression trend for these groups.

Canonical alternative splicing of primary RNA transcripts is an important biological process that allows a gene to have multiple RNA and protein isoforms with related or distinct functions. With more than 90% of the human genes undergoing alternative splicing, and a large fraction producing appreciable levels of two or more distinct populations of mRNA isoforms [36], we sought to determine the extent of difference between linear and candidate circRNA isoform diversity in our ovarian cancer samples. To estimate the linear transcript isoform abundances, RNA-seq data from our patient samples were aligned to the reference genome (GRCh38) in a paired end aware manner using tophat spliced read mapper [37]. Only concordant primary alignments were then taken in a subsequent step for transcript-resolution assembly and estimates of changes in gene expression using the cufflinks pipeline [38]. This filtering for concordant mappings on the genome ensured that read-pairs that contributed towards backsplice junction identification process are excluded for individual transcript isoform estimation and gene expression analysis. Cufflinks assembled a total of 63030 transcript isoforms from the merged samples with 13450 (21%) described as potentially novel isoforms belonging to 6272 known genes (Supplementary File S3). The high number of novel transcript isoforms reveals the highly uncharted state, complexity, and heterogeneity of the ovarian cancer transcriptome. We compared this diversity in linear transcripts with that of candidate circRNA isoforms (Figure 3A) and found that candidate circRNA isoforms are even more prevalent (wilcoxon test; p-value 2.324e-07) than their linear counterparts. The gene mucin 16 (MUC16), a cell surface associated protein and an ovarian cancer tumor marker [39–41] has a total of 11 detected linear transcript isoforms in our data with 4 of them described as potentially novel (Supplementary File S3) but at least 100 scrambled backsplice exon junctions that were also enriched in the RNAse R treated samples of OVCAR-3 cell-line. Almost all of the high abundance circRNA isoforms from this gene show an elevated expression in ovarian tumor compared to the peritoneum or lymph node metastatic sites of the three patients indicating an important role for these differentially regulated circRNAs in ovarian cancer progression. Therefore it appears that circRNA candidates have a rich diversity and differential regulation. Consequently, they could potentially influence the expression of thousands of genes in novel ways to regulate tumor growth and disease progression. Finally, to understand the relative abundances in terms of expression for linear and circRNA isoforms we compared the expression profiles of backsplice junction forming exons as a ratio of total read pairs supporting a circRNA form to the read pairs supporting the linear form for the two exons. We note that this analysis could in reality underestimate circRNA expression levels as it is hard to distinguish whether reads falling within an exon belong to a circular or linear transcript. However, in order to be more stringent with candidate circRNA expression, all such reads were taken as an evidence for linear transcript. The relative abundance of expression levels therefore gives an estimate for the usage of backsplice capable exons as linear or circRNA form. As shown in Figure 3B we found that most of the backsplice junction forming exons are primarily expressed as linear transcripts but that quite a significant fraction (5-10%) of these have a relatively higher abundance in the circRNA form. Further, Figure 3C shows a hierarchically clustered heatmap of the relative abundance levels of candidate circRNAs across the three patient samples and indicates a patient and tumor site specific expression pattern for candidate circRNAs. Hence it becomes imperative to identify the differential expression of circRNA candidates and delineate any circRNA biomarkers suitable for understanding ovarian cancer prognosis.

### Candidate circRNAs as potential miRNA sponges

**Figure 4:**
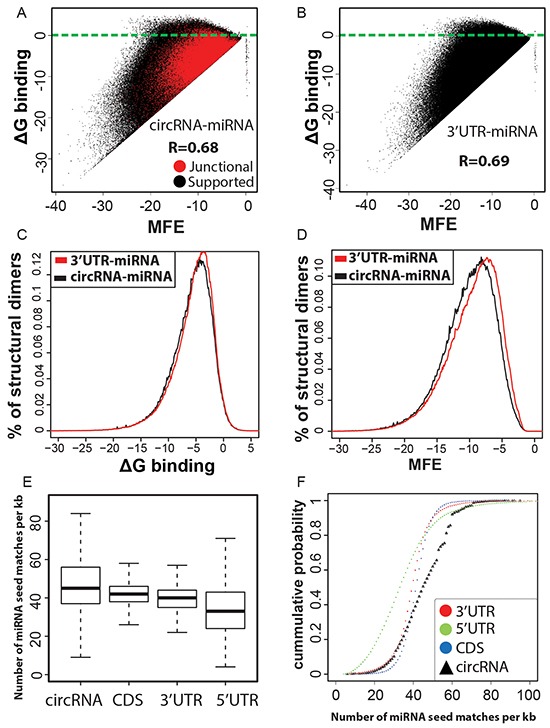
**A, B. Micro RNAs are predicted to interact with circRNAs.** Scatterplot of minimum free energy (MFE) versus binding energy (ΔG binding) for predicted structural dimers of circRNA-miRNA pairs are analogous to that of 3′UTR-miRNA seed matches, indicating the thermodynamic feasibility of these interactions at least similar to 3′UTR-miRNA binding events. A high correlation between MFE and ΔG binding for a vast majority of circRNA-miRNA cofolded dimers indicates favorable interaction. Only a marginal fraction (2%) of predicted interactions have ΔG binding >=0 (blue line separated). **C, D. circRNA-miRNA dimer cofolds are predicted to be more stable.** Binding energy distribution curves are similar for 3′UTR-miRNA and circRNA-miRNA structural dimers. The left shift of circRNA-miRNA curve for minimum free energy of dimer cofolds is highly significant (Wilcoxon rank sum test; p-value < 0.00001) and indicates more stability for circRNA-miRNA predicted secondary structures compared to 3′UTR-miRNA dimers. **E, F. circRNA candidates are enriched for miRNA seed matches.** Boxplots and Empirical cumulative distribution function (ECDF) curves showing a higher proportion of circRNA sequences having greater density of miRNA seed matches in comparison to 3′UTR, 5′ UTR regions and CDS.

MicroRNAs (miRNAs) are ~22nt long post-transcriptional regulators of gene expression that guide an effector silencing complex containing the Argonaut protein to the 3′ untranslated regions (3′ UTR) of target mRNAs resulting in their destabilization and translational repression [42–44]. There is a growing body of evidence that suggests the dysregulation of miRNA expression plays an important role in the development of a variety of human cancers [45, 46]. Recently, circRNAs have been implicated in the regulation of miRNA activity by behaving as competing endogenous sponge-like elements that antagonize the miRNA function [15, 22]. Therefore circRNAs seem to provide an additional layer of post-transcriptional control that help mRNAs escape miRNA mediated inhibition and hence have the potential to be used for RNA-based cancer therapies [24]. Consequently, we attempted to computationally determine the miRNA binding capabilities of our candidate circRNAs. As a first step we used Targetscan [47, 48] to identify the 8mer and 7mer sites that match the seed region of human miRNA sequences as obtained from miRBase [49], in the merged sequence of backspliced exons (circRNA) as well as UTR regions and coding sequences of genes. We then filtered the miRNA hits based on the context+ score and retained only matches that have a score of greater than or equal to −0.2 which is an indicator for stronger miRNA efficacy and hence reflects binding of miRNAs to their target sites [47]. In an additional refinement step for effective seed matches RNAcofold [50] was used to cofold and predict the secondary structures upon dimer formation between miRNA and subsequence of circRNA/UTR regions as obtained for consequential pairing from Targetscan. RNAcofold computes the secondary structure for the interacting RNA sequences that contributes a minimum of free energy (MFE) and also returns the net free energy change of binding interaction (ΔG) for the dimer cofold. As shown in Figure 4A & B, scatterplots of MFE and ΔG binding for circRNA-miRNA and 3′UTR-miRNA structural dimers follow similar distributions. This indicates thermodynamic feasibility for a majority of interactions between seed match containing subsequence of candidate circRNAs and miRNA mature sequences. Further, while ΔG binding frequency distribution curves are almost similar for 3′UTR-miRNA and circRNA-miRNA structural dimers, MFE scores of circRNA-miRNA duplexes in general fall in lower energy bands than 3′UTR-miRNA dimers (Figure 4C & 4D). These results could indicate candidate circRNAs are as potent as 3′UTRs to compete for miRNAs. However, when bound to circRNAs, miRNAs confer an energetically more stable state and this reinforces the idea of circRNAs acting as potential sinks for miRNAs. Finally, we also found a significantly (p-value < 0.00001) high density of miRNA seed matches with ΔG binding less than zero, indicating thermodynamic feasibility, for candidate circRNAs compared to 3′UTR and 5′ UTR sequences (Figure 4E & 4F). This greater enrichment for potentially effective miRNA seed matches in candidate circRNAs suggests a possible increased interaction due to cooperative effects from multiple target sites [51]. This could enhance the capabilities of circRNAs to alter normal miRNA activity.

### Differential expression of circRNAs between primary ovarian tumor and metastases

**Figure 5:**
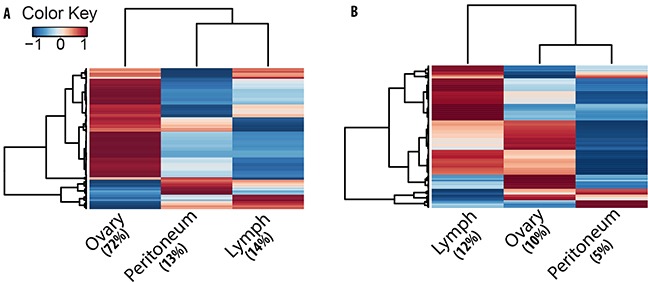
Clustering analysis indicates tumor stage specific expression trend for circRNAs Heat plots of **A.** circRNA and **B.** mRNA expression in tumor lesions. Each line in the plots is an isoform with colors indicating average expression (Z-score of log2-cpm values) from three patient samples for ovary, peritoneum and lymph node. Compared to mRNA, a higher fraction of circRNAs show tumor stage specific expression trend.

Gene expression products in ovarian cancer could represent candidate biomarkers and potential oncogenic factors involved in tumorogenesis and disease progression. We analyzed gene expression for both mRNAs and circRNAs and evaluated their abundances in an integrated manner to uncover crosstalk between networks of circRNAs, miRNAs, and mRNAs. Gene expression was estimated after aligning the RNA-seq data to reference genome (GRCh38) in a paired end aware manner using tophat spliced read mapper [37] and only keeping the concordant primary alignments. For each gene, expression was then quantified at the transcript level as the sum of paired-end fragments, and excluding any chimeric fragments using the featureCounts package [52]. Similarly, expression for circRNA candidates was also summarized as the aggregate total number of paired-end fragments that mapped to a scrambled exon junction in orientation supporting a head-to-tail backspliced junction instead of linear one and included both junctional and supporting read pairs. Finally, using three patient samples as replicates, differential expression was estimated between primary ovarian tumor and metastatic lesions using edgeR package [53] from Bioconductor (http://www.bioconductor.org/). For estimating significant differences, low expression loci were filtered out and only loci that were expressed at a minimum of three read counts in atleast three of the samples were taken for differential expression analysis. This reduced the number of pre-filtered genes and circRNAs for differential expression analysis to 2166;2683 for ovary vs peritoneum and 2803;2613 for ovary vs lymph node respectively. Table 2 gives the number of differentially expressed genes (Supplementary File S4) and circRNA candidates (Supplementary File S5) at a false discovery rate of less than 2% with at least 2-fold changes in expression. As reported in the table, the number of differentially expressed genes is less than one quarter of the number of differentially expressed circRNA candidates. This apparent large disparity between linear and circRNA differential expression is primarily due to a heterogeneous cancer transcriptome which often shows less reproducibility between patients and therefore masks any expression differences between the samples [7]. Nonetheless, circRNAs have a greater intracellular stability [21] due to their resistance to RNA exonucleases; therefore, they exhibit a robust expression pattern and may thus be more suitable candidates in identifying differences between primary tumor and metastatic lesions. This is further demonstrated in our hierarchical clustering analysis of average circRNA and mRNA expression for three patients (Figure 5). While as circRNA shows distinct expression clusters for primary tumor and metastatic lesions, mRNA expression is fuzzier between the tumors. This indicates the presence of a wide array of circRNA biomarkers potentially useful for disease screening and diagnosis.

**Table 2:** Number of differentially expressed genes and circRNA candidates between primary ovarian tumor versus peritoneal and lymph node metastases lesions

	Ovary/Peritoneum		Ovary/Lymph	
	mRNA	circRNA	mRNA	circRNA
Up	74	490	250	568
Down	51	786	217	623

**Figure 6:**
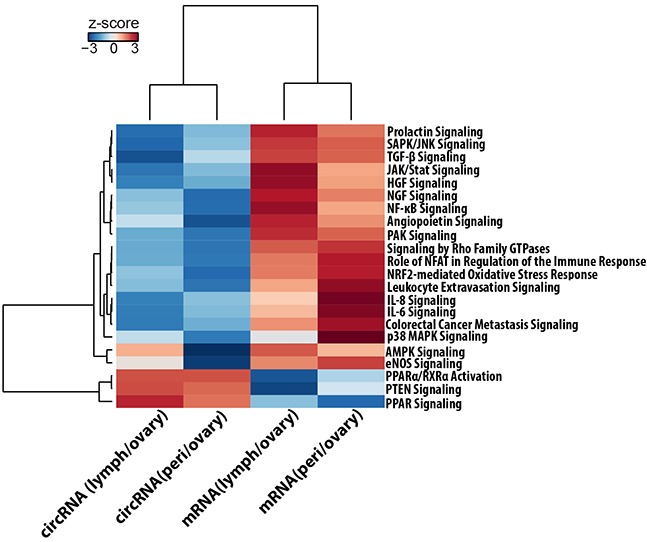
Ingenuity® Pathway Activity Analysis for circRNA and mRNA expression trends The figure represents a hierarchically clustered heatmap of the Pathway activity z-score which predicts the direction of change for a biological function or process. A positive z-score predicts that the biological process or disease is trending towards an increase and a negative score reflects a decrease. Signaling pathways that are typically over expressed for mRNA in metastatic tumors are downregulated for circRNAs.

Further, the canonical pathways analysis by Ingenuity's IPA toolkit (IPA^®^, QIAGEN Redwood City, www.qiagen.com/ingenuity) revealed an overwhelming enrichment of differentially expressed circRNA genes for cancer related diseases (Table 3). A merged representation of the top circRNA gene networks implicated in the “cancer” category is shown in Supplementary Figure S2 and demonstrates the central roles of TGF-β, NF-κB, ILK, PI3K/AKT and VEGF signaling biochemical pathways. We also compared the circular and linear expression for different cancer related biochemical pathways to identify the activation or inhibition expression trends of these pathways between metastatic lesions and primary tumor (Figure 6). We found that though most of these pathways are activated for linear RNA expression in metastases compared to primary site of origin, they are typically inhibited for circRNA expression and show exactly the opposite trend of the linear RNA. This points towards a system of an intricate interplay between the circular and linear expression in which circRNA and mRNA are tightly competing with each other for expression.

**Table 3:** Genes encoding differentially regulated circRNAs are highly enriched for cancer related diseases. The p-values indicate non-random association and have been calculated using the right-tailed Fisher Exact Test

Diseases or Functions Annotation	p-Value	# Genes
Abdominal Neoplasm	3.32E-43	1097
Epithelial Cancer	5.62E-39	1084
Urogenital Cancer	8.27E-36	648
Proliferation of cells	1.06E-40	571
Adenocarcinoma	4.44E-36	865
Pelvic cancer	3.19E-30	616
Genital tumor	3.86E-33	593
Migration of cells	4.97E-24	309
Genital tract cancer	1.11E-31	570
Female Genital neoplasm	5.07E-29	524
Breast or Ovarian Cancer	3.63E-24	312

Finally, as multiple circRNAs could contain binding sites for microRNAs, and those miRNAs in turn can target multiple genes, we considered a many-to-many relationship data-model for interaction networks between circRNAs, miRNAs and mRNA, and computed an average weighed expression (Average E_circ_) of differentially expressed circRNAs for each of the potential target genes (see Methods). These potential gene targets were obtained through microRNA target filter module of QIAGEN's Ingenuity^®^ Pathway Analysis (IPA^®^, QIAGEN Redwood City, www.qiagen.com/ingenuity) application as either experimentally observed or high confidence predicted targets of miRNAs and are highly enriched for cancer related pathways. This resulted in identifying 233 and 246 differentially expressed circRNAs containing binding sites for 1016 and 1070 miRNAs, and associated with 1140 and 1572 downstream gene targets in primary ovarian tumor versus peritoneal or lymph node metastases respectively (Supplementary File S6). These downstream gene targets display a positive correlation of expression with circRNAs predicted to bind the targeting miRNA (Supplementary Figure S3). Moreover, these downstream targeted genes are highly enriched for cancer associated pathways, indicating a functional role for circRNAs to potentially act as cancer drivers or suppressors.

## DISCUSSION

This work presents the first investigation of circular RNAs in clinical ovarian cancer tumors. Our targeted approach of searching for head-to-tail backspliced junctions from annotated exons systematically identified thousands of circRNA candidates in primary ovary tumor and matched peritoneum and lymph node metastases. This includes many novel genes in addition to other previously reported genes like HIPK2/3, ZKSCAN1 etc. capable of producing abundant circular RNA forms. The combined use of junction spanning and supportive reads as evidence for backspliced junctions conferred additional statistical power to the discovery and differential expression analysis of candidate circRNAs. Digestion of total RNA from SKOV3 and OVCAR3 cell lines with exoribonuclease enzyme RNase R showed an overwhelming enrichment for the identified backspliced junctions compared to linear mRNA, demonstrating the ability of our detection pipeline to recognize legitimate circRNAs.

Our method of detecting circular RNAs – looking for evidence of backspliced junctions – does not reveal the whole sequence of a circRNA. Although the existence of circRNAs has mainly been established by the presence of backspliced junction, the structure of circRNAs is largely unknown. In a few examples, the entire circle has been sequenced, and found to correspond exclusively to exonic sequences [54]. It is likely, therefore, that the majority of circRNAs detected by a non-canonical exon junction pair actually contain all the exonic sequences between the pair. Recent high-throughput analysis of RNase-R depleted RNA samples has shown enrichment for the exons expected in circRNAs, yet failed to find much if any enrichment for the corresponding introns [14]. This suggests that most circRNAs indeed consist of the backspliced exons plus all intervening exons but no introns. It is uncertain how widely this holds, or how the structure of circRNAs may vary under different conditions in particular in the context of neoplastic disease. In order to begin investigation of circRNA structure, we performed Sanger sequencing on selected circRNAs we had detected in cell lines. Using divergent primers centered on either the upstream or downstream exon, we used reverse transcriptase to generate linear cDNA corresponding to the sequence of circRNAs. Because the reverse transcriptase transcribes all the way around the circle, and continues with strand displacement for an additional 50 bp (or more), only cDNA generated from circRNAs will have the same sequence repeated, allowing it to be selectively amplified with primers in a PCR reaction. Additionally, by using either the forward or reverse primers in the reverse transcription step, we can identify whether the circRNA is in the sense or antisense strand. Although the cDNA of circles are too long to be sequenced in a single step by Sanger sequencing, use of multiple primers allowed sequencing of almost the entire sequence of circRNA from NFATC3 gene (Figure 2). In each of the four circRNAs studied this way from genes ARHGAP5, SPECC1, NFATC3, RANBP9, we found that the sequence of the circRNAs corresponded exactly to the spliced mRNA format, with the exception of the circularization. Thus in these few examples we have confirmed the structure of circRNAs predicted from the short, paired-end alignments through our detection pipeline.

We further show that genes containing multiple exons can produce alternate circular isoforms and there is a significantly higher diversity of circRNA isoforms in ovarian cancer compared to linear RNA. However, most of the circular forms are expressed at a low abundance and only around 10-15% are expressed at levels comparable to that of the linear RNA. Recently, it has been shown that circRNA biogenesis competes with pre-mRNA splicing and the circularization is dependent on flanking intronic complementary sequences harboring inverted ALU repeats [14, 18–20]. This association with repeat elements lends a dynamic nature to the circularization process as many of these sequences are highly susceptible to rapid evolutionary changes. Thus circularization efficiency is regulated through competition for RNA pairing between complementary intronic sequences and the alternative formation of inverted repeat Alu pairs lead to alternative circular isoforms from a single gene locus [19]. We observed a vast majority of our identified circRNA candidates (86%) containing a transposable element sequence (SINE/LINE) in the flanks of backsplice forming exons (Supplementary Figure S4) that could in-part explain the presence of many low frequency circular isoforms from multi-exon genes. In addition, enhanced transcription of oncogenes and other transcription factors in cancer cells [55, 56] could lead to inefficient canonical splicing due to an accelerated polymerase activity. This may also give rise to many of the low abundance circular structures as biogenesis of circRNAs has been shown to be negatively correlated with splicing efficiency [18].

We also report that a larger number of circRNAs are significantly differentially expressed between tumor types than mRNA. This is possibly due to a greater intracellular stability that results in robust expression pattern, for circRNA, across different patients and tumor stages. This makes them appropriate candidates for pinpointing differences in a highly heterogeneous ovarian cancer transcriptome and could offer a more robust classifier, for tumor subtypes or provide biomarkers for disease screening and diagnosis. Further, the genes encoding differentially expressed circRNAs are enriched for cancer associated as well as cell adhesion signaling pathways. This indicates a causal connection for circRNA differential expression in cancer development and progression. We found that signaling pathways that are typically over expressed for mRNA in metastatic tumors [57] are downregulated for circRNAs. Proliferative signaling pathways like PI3k/AKT, JAK/STAT and signaling by Rho GTPase were activated for linear mRNA transcripts and down regulated for circular RNA in metastases. Signaling pathways that are essential for epithelial to mesenchymal transition like NFkB, TGF beta, ILK [58, 59] were also activated for mRNA and inhibited for circRNA expression. Similarly, proinflamatory cytokines like IL6 and IL8 [60] and angiogenesis pathways like VEGF, ILK signaling and HGF signaling also showed upregulation for linear RNA but downregulation for circRNA expression in metastases. Inactivation of PTEN signaling leads to carcinogenesis and we observed a downregulation in mRNA transcripts for PTEN and PPAR signaling pathways and a corresponding upregulation of circRNA forms in metastases. These results show a trend that exhibits an inverse correlation between circRNA and mRNA expression for these pathways. It thus appears that RNA circularization modulates linear splicing and circRNA and mRNA expression are tightly competing with each other for splice sites. Our study shows a better consistency of circRNA expression compared to mRNA in high-grade tumors. It is possible that heterogeneous microenvironment of the tumors, especially, that of the lymph node metastasis could contribute to some of the observed differences in our results. Nevertheless, we anticipate the robustness of the circRNA expression with larger patient cohort would help in uncovering the molecular signatures that affect microenvironment changes in metastatic tumors.

Furthermore, circRNAs have been shown to harbor multiple binding sites for microRNAs [15, 22], and an miRNA in turn can alter the expression of hundreds of genes. For example, let-7 negatively regulates expression of some oncogenes like RAS and MYC as well as many other cell cycle progression genes [61, 62]. Reduced expression of Let-7 has been observed in various types of cancers, such as breast, lung and prostate cancers. The downregulation of let-7 correlates with increased lymph node metastasis and proliferation, suggesting a potential tumor suppressive role for this family of miRNAs in cancer progression [63, 64]. Similarly expression of miR-24 has been shown to enhance breast cancer tumor growth, local invasion and metastasis [65]. Our data indicates a reduced expression for both miR-24 and let-7 in primary ovarian tumor compared to peritoneal metastasis (Supplementary Figure S5). Differentially regulated circRNA candidates from several different genes, containing multiple binding sites for mir-24/let-7 show an elevated expression in ovarian tumor, providing a plausible explanation for the inhibition of these miRNAs in primary tumor compared to metastatic lesions. This also includes a circRNA candidate isoform from NFATC3 gene formed by second and third coding exons, for which the backsplice junction has been additionally validated through qPCR assay and Sanger sequencing (Figure 2). Therefore, with their potential to alter expression of oncogenic or tumor suppressor miRNAs, circRNAs could play profound roles in fine tuning the regulatory balance of miRNAs leading to cancer development, progression and metastases. Our results indicate an intricate interplay between networks of circRNAs, miRNAs and mRNAs in which a single circRNA could regulate many downstream genes via a common microRNA target. We observed co-expression of many of these circRNAs with their downstream gene targets that are highly enriched for cancer associated pathways, indicating a functional role for circRNAs to potentially act as cancer drivers. These emerging roles of circRNAs to communicate via miRNA prompts for new exciting opportunities in research to uncover the complex biological cross-talk, their role in carcinogenesis and efficacies as biomarkers for ovarian cancer diagnosis and prognosis.

## MATERIALS AND METHODS

### Study approval

All samples were collected at the department of Gynecologic Oncology at the Institut Claudius Regaud. The project was reviewed and approved by the Institut Claudius Regaud Human research Ethics Committee. All patients included in the study gave informed written consent prior to surgery.

### Sample collection

Three patients undergoing ovarian cancer treatment at the Institut Claudius Regaud, Toulouse were recruited for this study. All were diagnosed with Stage IIIC ovarian cancer with observed metastasis in the lymph node and peritoneum. During cytoreductive surgery, tissue samples from the ovary, peritoneum and lymph node were collected and frozen. Histology of the tumor sites indicates at least 80% purity. Although involvement of immune invasion or microenvironmental heterogeneity of the tumors cannot be ruled out, especially that for the lymph node metastasis but state of care surgery was used for tumor resection and samples are expected to contain mainly tumor cells.

### Cell culture

Ovarian cancer cells OVCAR3 and SKOV3 obtained from American Type Cancer Culture (ATCC) were cultured in DMEM (Gibco, Life technologies) supplemented with 10% FBS (Sigma), 1% antibiotic - Antimitotic solution (Gibco, Life Technologies). RNA isolations were performed after culturing the cell lines for at least one month using these standard conditions.

### RNA collection and RNase R enrichment

Total RNA was isolated using Qiagen RNAeasy mini kit according to manufacturer's protocols including oncolumn DNAse digestion. For RNase R treatment, 2 micrograms of total RNA was briefly heated to 70°C to denature, and then cooled to 40°C on a thermo cycler. 20units of RNAse R (Epicenter) and 1 unit/microliter murine Ribonuclease Inhibitor (New England Biolabs) were added to the denatured RNA samples and incubated at 40°C for 1 hr.

### Library preparation and sequencing

100 ng of total RNA of SKOV3 and OVCAR3 cells treated with RNAse R (as described above) and untreated samples were used for library preparation. cDNA synthesis was done using Ovation RNA-Seq system v2 (NuGEN) with SPIA amplification. RNA-Seq libraries were prepared by using Truseq library preparation protocol (Illumina). Sequencing was performed on an illumina Hiseq instrument with 100bp paired end reads.

### Quantitative real-time PCR

Total RNA was isolated from SKOV3 and OVCAR3 cells. cDNA synthesis was done by using First Strand cDNA Synthesis Kit for RT-PCR (AMV) from ROCHE using either random hexamer or oligo dT as indicated. Primers used in qRT-PCR were designed as convergent primers to detect circular junctions, and to cross the backsplice junction (Supplementary File S1). Real-time PCR was carried out in triplicate on **StepOnePlus™** Real-Time PCR Systems (Life Technologies). Quantification of circRNA enrichment in comparison with linear housekeeping genes like Beta actin (ACTB) and GAPDH is also done. For confirmation of circular junctions a PCR reaction was performed using one step RT PCR kit (Qiagen) with convergent circ Primer pair was used for template amplification.

### Sanger sequencing

For each of the four circRNAs to be sequenced, primers were designed in regions about 50 bp to either side of the junction, with one primer pair on each of the exons in the noncanonical junction. Each primer pair was divergent, overlapping by only 6 bp, and a Tm of about 60°C. Reverse transcription was carried out on pooled OVCAR RNA using Thermoscript RT (Life Technologies) according to directions, with a pool of the four primers that would amplify template strand circRNAs. The resultant cDNA was subjected to PCR amplification with each primer pair. The bands were gel purified and submitted for Sanger sequencing on a Applied Biosystems 3130xl Genetic analyzer, using each of the four primers designed against that circle.

### *In silico* detection of circRNA candidates from paired end RNA-seq data

A non-canonical mode of RNA splicing results in a scrambled junction, in which a downstream exon (Exon2) is “backspliced” in a head-to-tail fashion to an upstream exon (Exon1) into a circular RNA molecule placing Exon2 upstream of Exon1. We created a reference scrambled exome for all possible pairs of intragenic non-linear combinations of exons, as well as single exon “backsplices”, by either joining the 3′ and 5′ ends of a single exon or joining these ends from a downstream to an upstream exon. This represents the sample space for all possible exon-junction structures that can occur through circularization events. We aligned the RNA-seq data to this reference scrambled exome with Bowtie2 [25]. In a subsequent step of the circRNA detection pipeline, only the primary alignments were kept for further analysis and were filtered to remove any potential PCR duplicates with samtools rmdup [66]. Finally from these alignments, a scrambled junction is inferred as a junctional circRNA candidate when one mate of a paired-end read aligns at the junction with a minimum of 10bp overlap with either exon and the other mate aligns at either Exon2 or Exon1. Alternatively, in absence of a direct junctional read, supportive evidence is also considered when mates of a paired-end read align to the exons in orientation suggesting a scrambled junction instead of a linear junction (Figure 1A).

### Differential expression (circRNA)

For each circRNA candidate, expression was summarized as the aggregate total number of paired-end fragments that mapped to a scrambled exon junction in an orientation supporting a head-to-tail “backsplice” instead of a “linear splice”. We included both reads spanning actual junctions (“junctional”) and divergent paired-ends that supported (“supported”) a circular structure. Finally considering samples from three patients as biological replicates statistical significance of differential expression was estimated at a positive predictive value of 98% (FDR < 0.02) with a minimum of two fold change in expression between primary ovarian tumor and metastatic lesions, using edgeR package [53] from Bioconductor (http://www.bioconductor.org). For estimating significant differences, only loci that were expressed at reasonable levels of a minimum of three read counts in atleast three of the samples were taken for differential expression analysis. Before estimating the differential expression, raw count data was normalized for variation in sequencing depths between the samples and adjusted for RNA composition effects using the default Trimmed Mean of M-values (TMM) method. A design matrix was then defined based on an additive linear model with patient as the blocking factor and estimates for trended and tagwise dispersion parameters were computed. Finally, a negative binomial generalized linear model was fitted to determine differential expression using the likelihood ratio test which computes p-value and the Benjamini-Hochberg adjusted p-values (FDR) for each tag and ranks them in order of evidence for differential expression.

### Differential expression (Genes)

Gene expression was estimated after aligning the RNA-seq data to reference genome (GRCh38) in a paired end aware manner using tophat spliced read mapper [37] and only keeping the concordant primary alignments. These were again filtered to remove any potential PCR duplicates with samtools rmdup [66]. For each gene, expression was then quantified at the transcript level as the sum of paired-end fragments, and excluding any chimeric fragments using the featureCounts package [52]. Differential gene expression was estimated in a similar manner as described above for circRNAs by considering the three patients as biological replicates and identifying genes with at least two fold changes in expression at an FDR of < 0.02 using edgeR.

### Average E_circ_

Weighted Expression of circRNAs targeting a given microRNA (miRNA-x) is calculated as,

$$E_{\text{circ}}=\frac{\sum_{i=1}^{N}n_{xi}x_{i}}{N}$$

Where *N* is the number of circRNAs targeting miRNA-x, *n_xi_* the number of seed matches on i^th^ circRNA for miRNA-x and *x_i_* is the fold change expression of the i^th^ circRNA. An Average E_circ_ is then computed for each of the downstream target genes that shows either an experimentally observed interaction with miRNAs or contains highly significant predicted binding sites for miRNAs.

*Average E_circ_ = Average* (*E1_circ_* + *E2_circ_* + … + *EN_circ_*)

### Data access

The data for cell-lines has been submitted to Sequence Read Archive (NCBI SRA) under accession numbers SRR1772957, SRR1772257, SRR1777309 and SRR1777310.

## SUPPLEMENTARY FIGURES AND TABLES














