# Supplementary material for: Altered expression pattern of circular RNAs in primary and metastatic sites of epithelial ovarian carcinoma
Source: Oncotarget. 2016 Apr 22;7(24):36366–81. doi: 10.18632/oncotarget.8917 (PMC5095006; doi:10.18632/oncotarget.8917)
Supplement: Supplementary file 1 [file oncotarget-07-36366-s001.pdf]

## SUPPLEMENTARY FIGURES AND TABLES

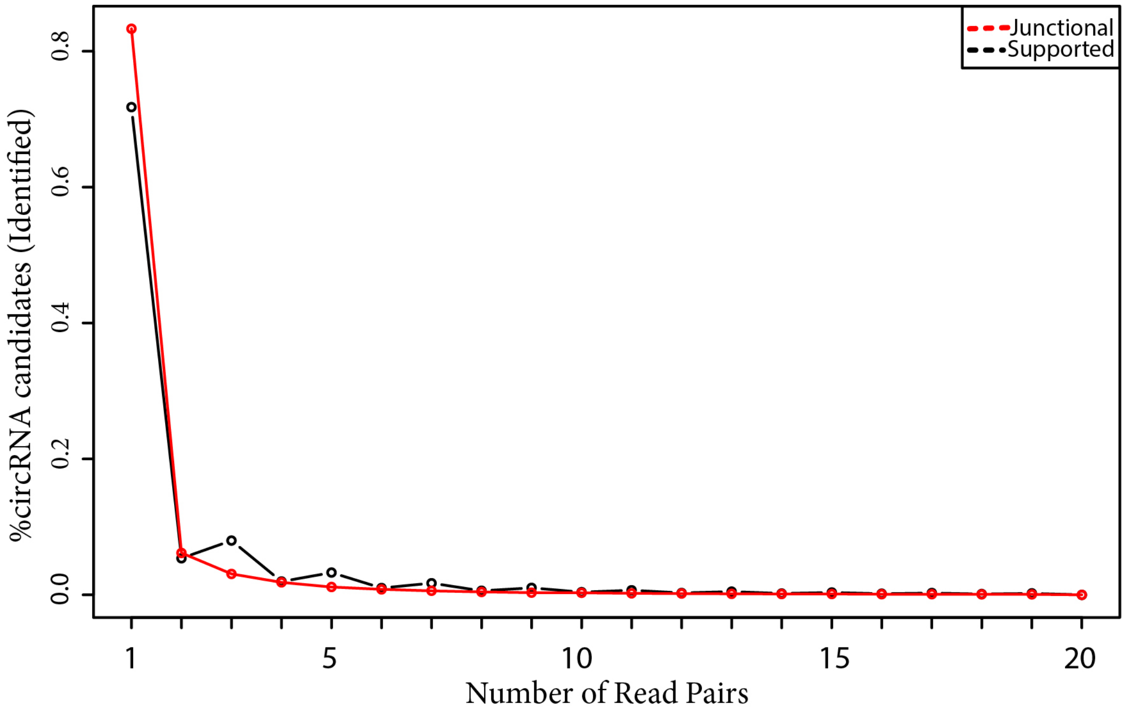


**Supplementary Figure S1: Plot shows the percentage of initially identified backsplice junctions against the Read depth value.** Junctional circRNA candidate have one mate of a paired-end read aligning at the junction with a minimum of 10bp overlap with either exon. Supported candidates only have read pairs where mates of the pair align to exons in orientation suggesting a backspliced junction instead of a linear junction. Most preliminary identified backsplices are supported by only one read pair, indicating an experimental artifact source of either template switching, chimeric amplification or trans-splicing, which are rare and random events, not expected to produce abundant products. We discarded all the initially identified backsplices having less than three junctional or less than ten non-junctional read pairs supporting the backsplice exon junction.

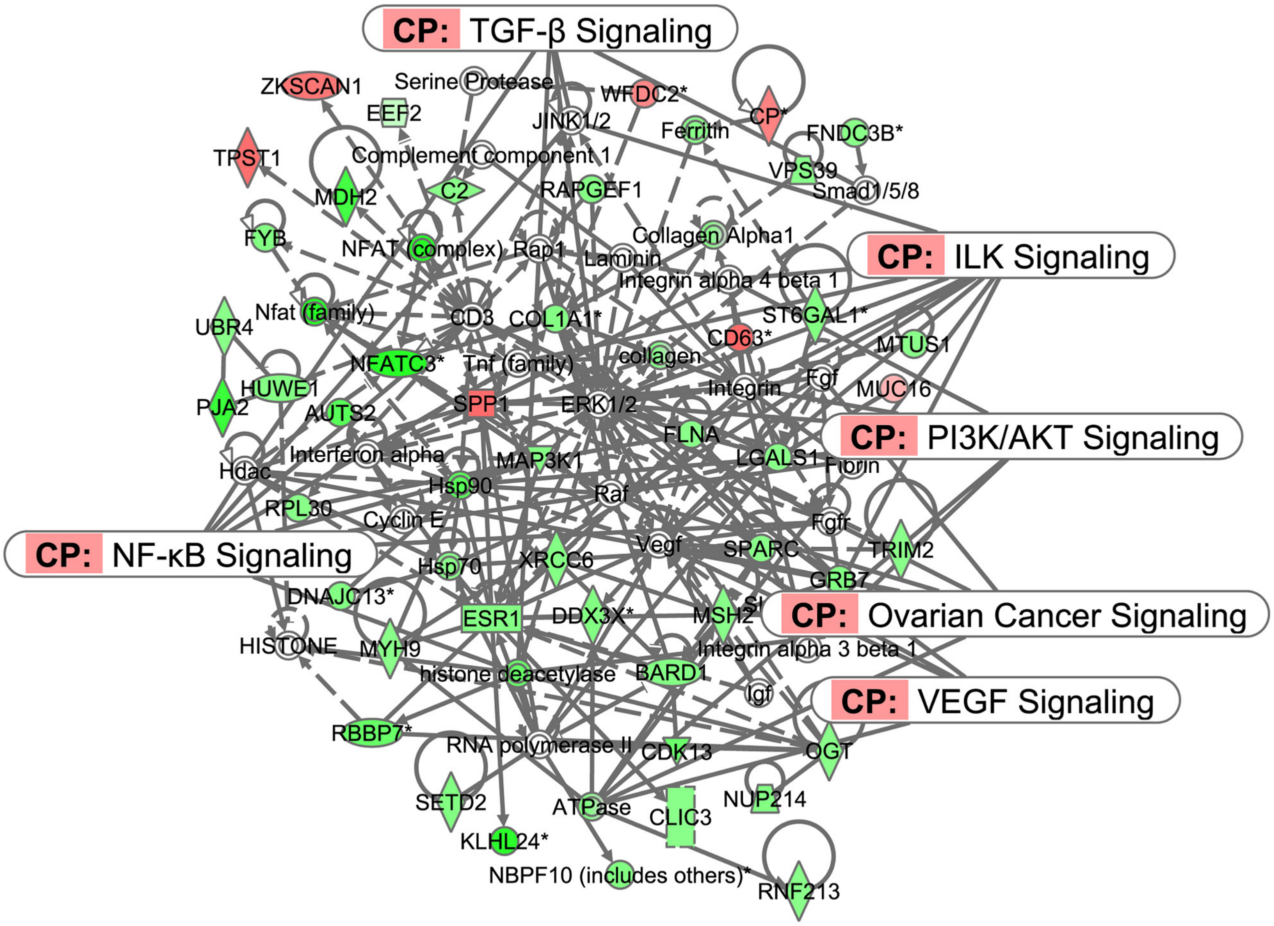


**Supplementary Figure S2: Regulatory network of differentially expressed circRNA genes in peritoneum compared to primary site of origin.** Genes encoding differentially regulated circRNAs between peritoneal metastasis and primary tumor were used to construct the network. In the resulting network, nodes represent genes and the edges represent biological relationship between two nodes as supported by at least one reference from literature in the IPA® knowledge database. This regulatory network indicates a causal connection for differential expression of circRNAs in cancer related pathways towards ovarian cancer. Green shows downregulation and red color shows upregulation of circRNA expression in peritoneal metastasis. CP: Canonical Pathways.

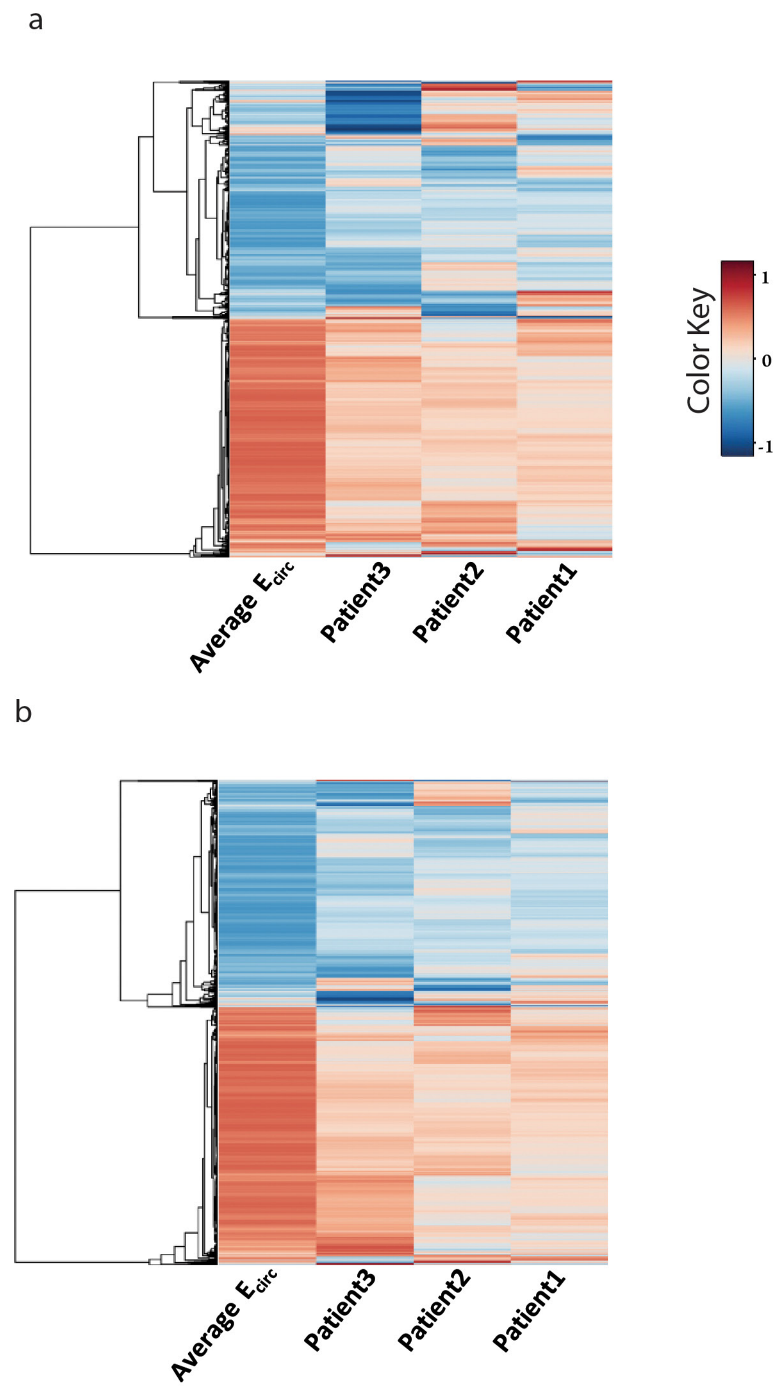


**Supplementary Figure S3: Differentially expressed circRNA candidates in primary ovarian tumor versus peritoneal metastasis a.** and ovarian tumor versus lymph node metastasis **b.** show co-regulation with their downstream gene targets. circRNAs usually have multiple sites for a single micro RNA which in turn can target multiple genes. Also, a single gene can have more than one targeting microRNA. We computed an average weighted expression of circRNAs for each of the potential target genes (*Average* $E_{circ}$). A hierarchically clustered heatmap of the gene expression data along with the *Average* $E_{circ}$ expression from regulating circRNAs shows co-regulation of downstream target genes with differentially expressed circRNAs.

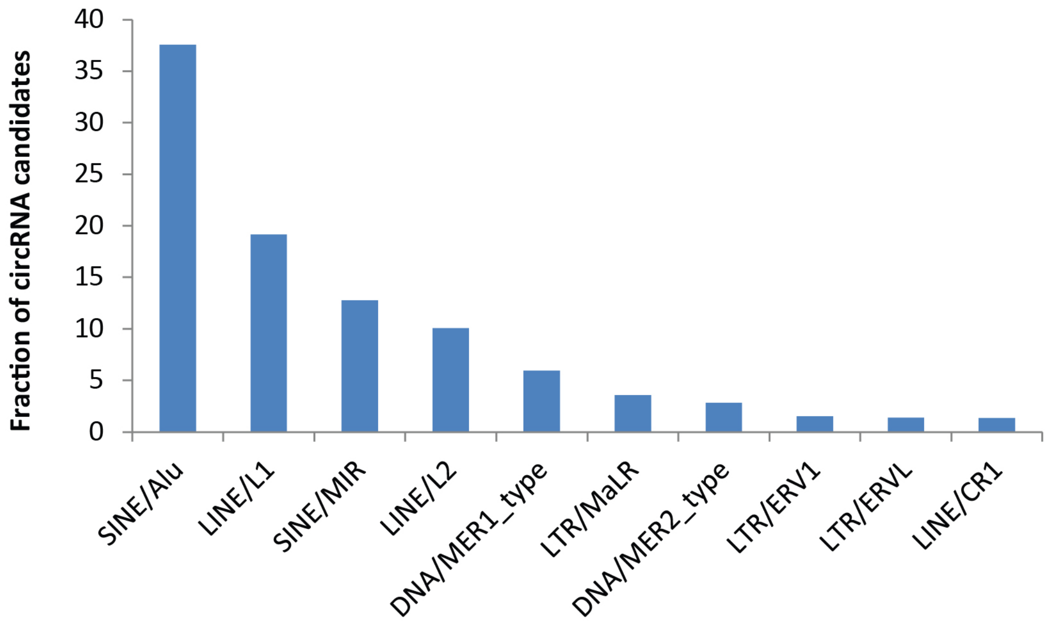


**Supplementary Figure S4: Identified circRNAs are associated with SINE/Alu repeat elements.** Large fraction of circRNA candidates (86%) contains a transposable fragment in the flanking sequenceof the backsplice capable exons with a dominant contribution from SINE and LINE superfamilies.

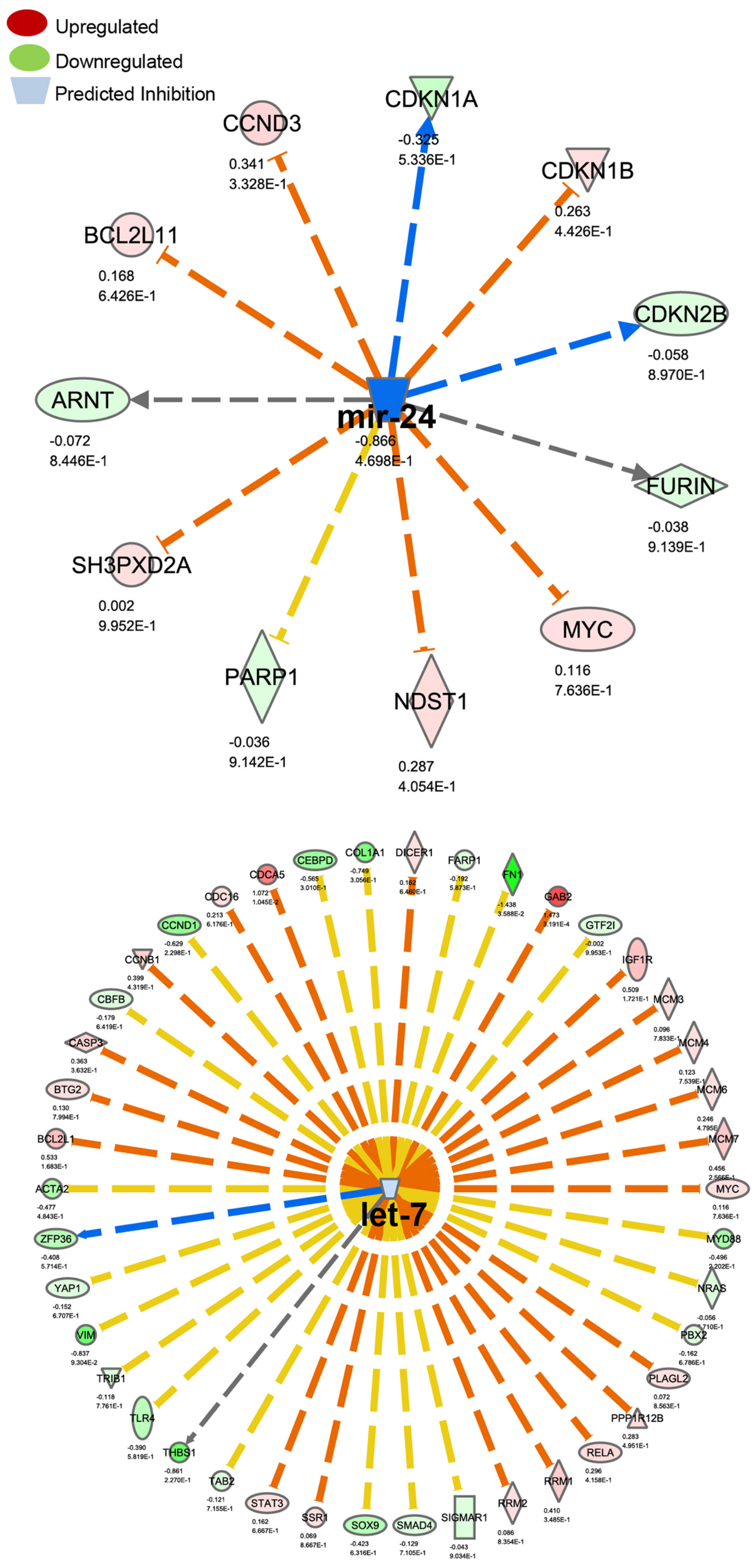


**Supplementary Figure S5: mir-24 and let-7 predicted to be downregulated in primary tumor.** QIAGEN's Ingenuity® Pathway Analysis (IPA®, QIAGEN Redwood City, www.qiagen.com/ingenuity) module predicts inhibition for upstream factors mir-24 and let-7 in ovarian tumor compared to peritoneal metastasis based on the gene expression state of their known downstream targets.

**Supplementary File S1: Primer sequences**

**See Supplementary File 1**

**Supplementary File S2: List of identified circRNA junctions**

**See Supplementary File 2**

**Supplementary File S3: Cufflinks assembled transcripts**

**See Supplementary File 3**

**Supplementary File S4: Differentially expressed genes**

**See Supplementary File 4**

**Supplementary File S5: Differentially expressed circRNAs**

**See Supplementary File 5**

**Supplementary File S6: MicroRNA binding sites for differentially expressed circRNAs**

**See Supplementary File 6**
